# Photoperiod Impacts Nucleus Accumbens Dopamine Dynamics

**DOI:** 10.1523/ENEURO.0361-22.2023

**Published:** 2023-02-15

**Authors:** Alexis N. Jameson, Justin K. Siemann, James Melchior, Erin S. Calipari, Douglas G. McMahon, Brad A. Grueter

**Affiliations:** 1 Neuroscience Graduate Program, Vanderbilt University, Nashville, TN 37232; 2 Vanderbilt Brain Institute, Vanderbilt University, Nashville, TN 37232; 3 Department of Molecular Physiology and Biophysics, Vanderbilt University, Nashville, TN 37232; 4 Vanderbilt Center for Addiction Research, Vanderbilt University, Nashville, TN 37232; 5 Department of Pharmacology, Vanderbilt University, Nashville, TN 37232; 6 Department of Biology, Vanderbilt University, Nashville, TN 37232; 7 Department of Anesthesiology, Vanderbilt University Medical Center, Nashville, TN 37232

**Keywords:** circadian photoperiod, dopamine, nucleus accumbens, sex differences

## Abstract

Circadian photoperiod, or day length, changes with the seasons and influences behavior to allow animals to adapt to their environment. Photoperiod is also associated with seasonal rhythms of affective state, as evidenced by seasonality of several neuropsychiatric disorders. Interestingly, seasonality tends to be more prevalent in women for affective disorders such as major depressive disorder and bipolar disorder (BD). However, the underlying neurobiological processes contributing to sex-linked seasonality of affective behaviors are largely unknown. Mesolimbic dopamine input to the nucleus accumbens (NAc) contributes to the regulation of affective state and behaviors. Additionally, sex differences in the mesolimbic dopamine pathway are well established. Therefore, we hypothesize that photoperiod may drive differential modulation of NAc dopamine in males and females. Here, we used fast-scan cyclic voltammetry (FSCV) to explore whether photoperiod can modulate subsecond dopamine signaling dynamics in the NAc core of male and female mice raised in seasonally relevant photoperiods. We found that photoperiod modulates dopamine signaling in the NAc core, and that this effect is sex-specific to females. Both release and uptake of dopamine were enhanced in the NAc core of female mice raised in long, summer-like photoperiods, whereas we did not find photoperiodic effects on NAc core dopamine in males. These findings uncover a potential neural circuit basis for sex-linked seasonality in affective behaviors.

## Significance Statement

Day length, or photoperiod, is a reliable indicator of the changing seasons, and is a powerful environmental cue influencing affective state and behavior. While studies have shown that photoperiod can modulate neurotransmitter systems involved in affective behavior, like the dopamine system, there are few studies examining effects of photoperiod on the synaptic level. Here we assess mesolimbic dopamine dynamics in mice maintained in seasonal photoperiods. We found a sex-specific effect wherein photoperiod modulates dopamine release from and uptake in dopaminergic terminals in the nucleus accumbens (NAc) core of female mice with long summer-like photoperiods increasing dopamine release, thus uncovering a potential synaptic basis for sex-linked seasonality in affective behaviors.

## Introduction

Circadian photoperiod, or day length, is an environmental signal that synchronizes daily biological rhythms to local time and induces seasonal changes in behavior and physiology (Øverland et al., 2019; Magnusson, 2000; Zhang et al., 2021; Green et al., 2015; Siemann et al., 2021). While seasons in the temperate zones are marked by a variety of environmental changes, photoperiod is the most reliable environmental cue that encodes seasons and allows organisms to predict and adapt to seasonal changes to their environment. The amplitude of seasonal change in photoperiod varies by latitude, as does the prevalence of seasonal mood disorders (Øverland et al., 2019; Rosen et al., 1990; Magnusson, 2000; Siemann et al., 2021; Zhang et al., 2021). Bright light therapy treats seasonal mood disorders by increasing the duration of light a person experiences in a 24-h day, effectively lengthening their photoperiod (Melrose, 2015). Identifying photoperiod-sensitive systems within brain regions relevant to affective behaviors will provide insight the regulation of affective and motivated behaviors.

Dopamine is a neuromodulator that plays a critical role in regulating affective state and state-dependent behaviors (Mogenson et al., 1980; Nestler and Carlezon, 2006; Floresco, 2015; Turner et al., 2018). More specifically, dopamine input to the nucleus accumbens (NAc) from the ventral tegmental area (VTA) has been implicated in the pathophysiology of mood disorders (Nestler and Carlezon, 2006; Floresco, 2015). Evidence suggests photoperiod can modulate dopamine in other brain regions as measured by tissue content (Carlsson et al., 1980; Goda et al., 2015), dopamine turnover (Itzhacki et al., 2018), transporter availability (Neumeister et al., 2001), and presynaptic dopamine synthesis (Eisenberg et al., 2010). To date, studies have not examined photoperiod’s effect on the dopamine system at the synaptic level.

Dopamine signaling in the NAc is tightly and dynamically regulated through both VTA neuron activity and intra-accumbal mechanisms at dopamine axon terminals (Sulzer et al., 2016; Nolan et al., 2020). VTA dopamine neurons display two functionally distinct firing modes. Shifts from low frequency tonic firing to phasic burst firing in these neurons encode information such as reward-prediction error and incentive salience (Ljungberg et al., 1992; Schultz, 1998, 2007; Tsai et al., 2009; Cohen et al., 2012; Kutlu et al., 2021). Dopamine release in the NAc is also regulated through local mechanisms including inhibition via presynaptic dopamine D2 autoreceptors as well as facilitation via presynaptic nicotinic acetylcholine receptors (Shin et al., 2017; Yorgason et al., 2017). Once released, clearance of dopamine from the synaptic space primarily occurs via re-uptake into dopaminergic terminals through the dopamine transporter (DAT; Giros et al., 1996; Jones et al., 1998), where it is then repackaged into vesicles for future release by vesicular monoamine transporter 2 (VMAT2; Erickson et al., 1992; Nolan et al., 2020). DAT activity shapes the kinetics of dopamine signaling and can influence dopamine release through the recycling of transmitter (Egaña et al., 2009; Condon et al., 2019). Furthermore, changes to NAc DAT function by inhibition, reversal, or genetic manipulation results in changes to goal-directed behaviors (Jones et al., 1998; Calipari et al., 2013, 2017; Sørensen et al., 2021), demonstrating how subsecond dopamine signaling dynamics powerfully influence behavior.

Sex differences in the function of the mesolimbic dopamine pathway are well established (Q.D. Walker et al., 2000; Dluzen and McDermott, 2008; Becker, 2009; Yoest et al., 2014; Calipari et al., 2017; Zachry et al., 2021), making it critically important to consider sex of the animal when assessing photoperiod effects on this system. VTA dopamine neuron activity, NAc dopamine release and uptake, DAT expression, and VMAT2 activity are all greater in females compared with males (Q.D. Walker et al., 2000; Dluzen and McDermott, 2008). Interestingly, several neuropsychiatric disorders that involve the dopamine system display seasonal patterns where seasonality is typically more prevalent in women (Magnusson, 2000; Kim et al., 2011; Geoffroy et al., 2014; Otte et al., 2016).

Here, we assess the effect of seasonal photoperiods on NAc dopamine dynamics in mice using fast-scan cyclic voltammetry (FSCV). We found a sex-specific effect wherein photoperiod modulates dopamine release and uptake in the NAc of female mice. Specifically, female mice raised in long summer-like photoperiods exhibit greater dopamine release, thus uncovering a potential synaptic basis that provides insight to sex-linked seasonality of affective behaviors.

## Materials and Methods

### Animals

We used male and female C3Hf^+/+^ mice for all experiments. C3Hf^+/+^ mice endogenously synthesize melatonin and lack the retinal degeneration alleles of the parent C3H strain and are therefore responsive to photoperiod manipulation (Ebihara et al., 1986; Goto et al., 1989; Tosini and Menaker, 1998; Contreras-Alcantara et al., 2012). Mice were bred and maintained in groups of two to five per cage under photoperiods we indicate here as Short (8 h of light 16 h of darkness), Equinox (12 h of light and 12 h of darkness), or Long (16 h of light and 8 h of darkness). Animals were continuously maintained in these photoperiod conditions from embryonic day 0 (E0) until they were used for experiments between postnatal day 50 (P50) and P90. FSCV experiments were conducted in NAc slices from Short, Equinox, and Long photoperiod mice. All tissues were isolated within a 2-h window of the middle of the light-phase of each light cycle. Lights off denoted zeitgeber time (ZT) 12; therefore, Short animals were used at ZT8, Equinox animals were used at ZT6, and Long animals were used at ZT4. Experiments were performed in accordance with Institutional Animal Care and Use Committee and National Institutes of Health guidelines.

### Fast-Scan cyclic voltammetry

*Ex vivo* FSCV was used to assess dopamine release and uptake dynamics in the NAc core of Short, Equinox, and Long photoperiod animals (*n* = 4–7 animals per sex and photoperiod group, using 1 brain slice per animal). Mice were humanely killed and the brain was rapidly removed. Using a Leica Vibratome, 250-μm-thick sagittal sections containing the NAc core were collected from whole brain tissue in oxygenated (95% O_2_; 5% CO_2_) artificial CSF (aCSF) containing (in mm): 126 NaCl, 2.5 KCl, 1.2 NaH_2_PO_4_, 2.4 CaCl_2_, 1.2 MgCl_2_, 25 NaHCO_3_, 11 glucose, and 0.4 L-ascorbic acid, and pH adjusted to 7.4. Slices were transferred to a chamber containing oxygenated aCSF. All experiments were performed using a Scientifica SliceScope Pro System in 32°C aCSF with a flow rate of 2 ml/min. The carbon fiber microelectrode (100−200 μm in length, 7-μm radius) and bipolar stimulating electrode were placed in close proximity in the NAc core. A single electrical pulse (750μA, 4 ms, monophasic) was applied to the tissue every 4 min to evoke dopamine release. We also evoked dopamine release using a train of five pulses at 5, 10, or 20 Hz (750 μA, 4 ms, monophasic). Extracellular dopamine was recorded by applying a triangular waveform (−0.4 to +1.2 to −0.4 V vs Ag/AgCl, 400 V/s).

For pharmacological experiments using DAT inhibitor, GBR 12783 (Tocris Bioscience/Bio-Techne), peak evoked dopamine release was collected in aCSF until a stable baseline was established (three collections with <10% variability) using a single electrical pulse every 4 min. GBR 12783 was bath applied to the slice at 300 nm, 1 μm, and 3 μm concentrations. We collected peak evoked dopamine release at each concentration until stable responding was achieved (three collections with <10% variability) before moving onto the next highest concentration.

### Voltammetry data analysis

Demon voltammetry and analysis software was used to analyze all FSCV data (Yorgason et al., 2011). Data collected using single electrical pulse were modeled using Michaelis–Menten kinetics using the kinetic analysis tool. By using Michaelis–Menten kinetic analysis, we derived parameters such as the peak dopamine concentration released following stimulation (amplitude of signal) and maximal rate of dopamine uptake by DAT (*V*_max_). As described previously (Wu et al., 2001; Yorgason et al., 2011; Calipari et al., 2017), *K_m_* from the Michaelis–Menten equation represents the affinity for dopamine binding to DAT. According to standard FSCV analysis methods (Yorgason et al., 2011), and based on previous research on the affinity of dopamine for the DAT (Wu et al., 2001), we set the *K*_m_ parameter to 160 nm for each animal and allowed *V_max_* to vary to determine baseline *V_max_*. For experiments using GBR 12783, we determined a baseline *V_max_* for each animal once stable baseline responding was achieved in aCSF as described above. For subsequent GBR 12783 concentrations, we held the baseline *V_max_* constant and allowed *K_m_* to vary to determine apparent *K_m_*. Apparent *K_m_* is the Michaelis–Menten constant as observed under conditions, such as competitive inhibition, that would impede determining its true value. Measuring apparent *K_m_* allows determination of the sensitivity of DAT to pharmacological competitive inhibition by GBR 12783. Raw data were calibrated in each data file before Michaelis–Menten modeling. Recording electrodes were calibrated by recording the peak current (nA) to a known concentration of dopamine (3 μm) via flow-injection system, and we used these values to convert current to dopamine concentration.

### Statistical analysis

Prism 9 (GraphPad Software Inc.) was used for all statistical analyses. For all analyses, α was set as 0.05, with *p* values < α indicating a statistically significant difference. Statistical significance was determined by two-way ANOVA. All *post hoc* analysis was performed using Tukey’s multiple comparison tests and standard error of the mean was used for all experiments. Power analyses were performed with preliminary data during the acquisition of each new data set. The sample size obtained from each power analysis calculation was then compared with sample sizes reported in the literature for similar experiments. Errors bars depicted in figures represent SEM.

## Results

### Long, summer-like photoperiod increases evoked dopamine release in the NAc core

**Figure 1.**
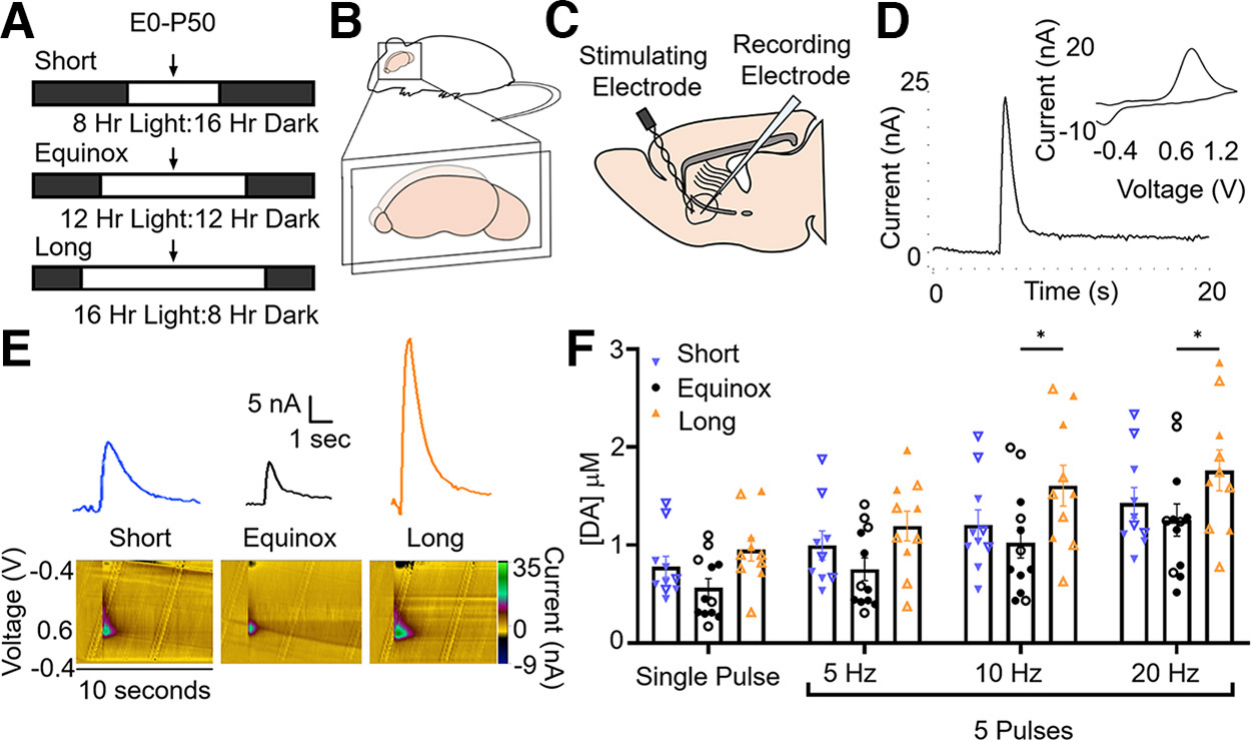
Long photoperiod increases evoked dopamine release in the NAc core. ***A***, Light cycles for Short, Equinox, and Long photoperiod conditions exposed to mice from embryonic day 0 (E0) until they were used for voltammetry at postnatal day 50 (P50). ***B***, Sagittal slices containing the NAc core were collected from each animal. ***C***, Placement of stimulating and recording electrodes in the NAc core. ***D***, Representative current trace and voltammogram. ***E***, Representative traces and color plots for mice raised in Short, Equinox, and Long photoperiods. ***F***, Evoked NAc core dopamine release across tonic and phasic frequencies in male (open symbols) and female (closed symbols) mice raised in Short (*N* = 9), Equinox (*N* = 12), and Long (*N* = 10) photoperiods. Main effect of photoperiod (*F*_(2,115)_ = 10.43, *p* < 0.0001), stimulation frequency (*F*_(3,115)_ = 13.18, *p* < 0.0001). *p < 0.05 for Long versus Equinox. Data were analyzed using two-way ANOVA and Tukey’s *post hoc* multiple comparisons test with an α value of *p* = 0.05. Data are represented as mean ± SEM.

We measured electrically evoked DA release from the NAc core in *ex vivo* slices from both male and female mice raised in Short (8L:16D), Equinox (12L:12D), or Long (16L:8D) photoperiods (Fig. 1*A–C*). The representative cyclic voltammogram (Fig. 1*D*), shows the characteristic redox signature of dopamine. Electrical stimulation evoked clear and distinct transient dopamine release and uptake events that were time-locked to stimulation with current peaks at the oxidation (0.6 V) and reduction (−0.2 V) voltages for catecholamines (Fig. 1*E*). Peak dopamine release concentration was calculated from the peak height of the FSCV response using calibrated electrodes, with sample sizes as follows: Short (*N* = 9), Equinox (*N* = 12), and Long (*N* = 10) where N is number of animals. To assess whether photoperiod differentially affected dopamine release from tonic and phasic firing modes of dopamine neurons projecting to the NAc core, we used a range of stimulation frequencies from single pulse to 20 Hz. Two-way ANOVA revealed that increasing stimulation frequency increased dopamine release in all photoperiod groups, as expected (Sulzer et al., 2016; Nolan et al., 2020; *F*_(3,115)_ = 13.18, *p* < 0.0001; Fig. 1*F*). Photoperiod significantly affected the magnitude of evoked dopamine release (*F*_(2,115)_ = 10.43, *p* < 0.0001). Specifically, Tukey’s *post hoc* multiple comparisons test showed that dopamine release was elevated in NAc core from Long photoperiod mice compared with NAc core from Equinox photoperiod mice in the 10-Hz (*p* = 0.018) and 20-Hz (*p* = 0.046) conditions. Taken together, our results indicate that Long summer-like photoperiods can enhance evoked dopamine release in the NAc core.

### Effects of long, summer-like photoperiod on dopamine release and uptake rate are female specific

**Figure 2.**
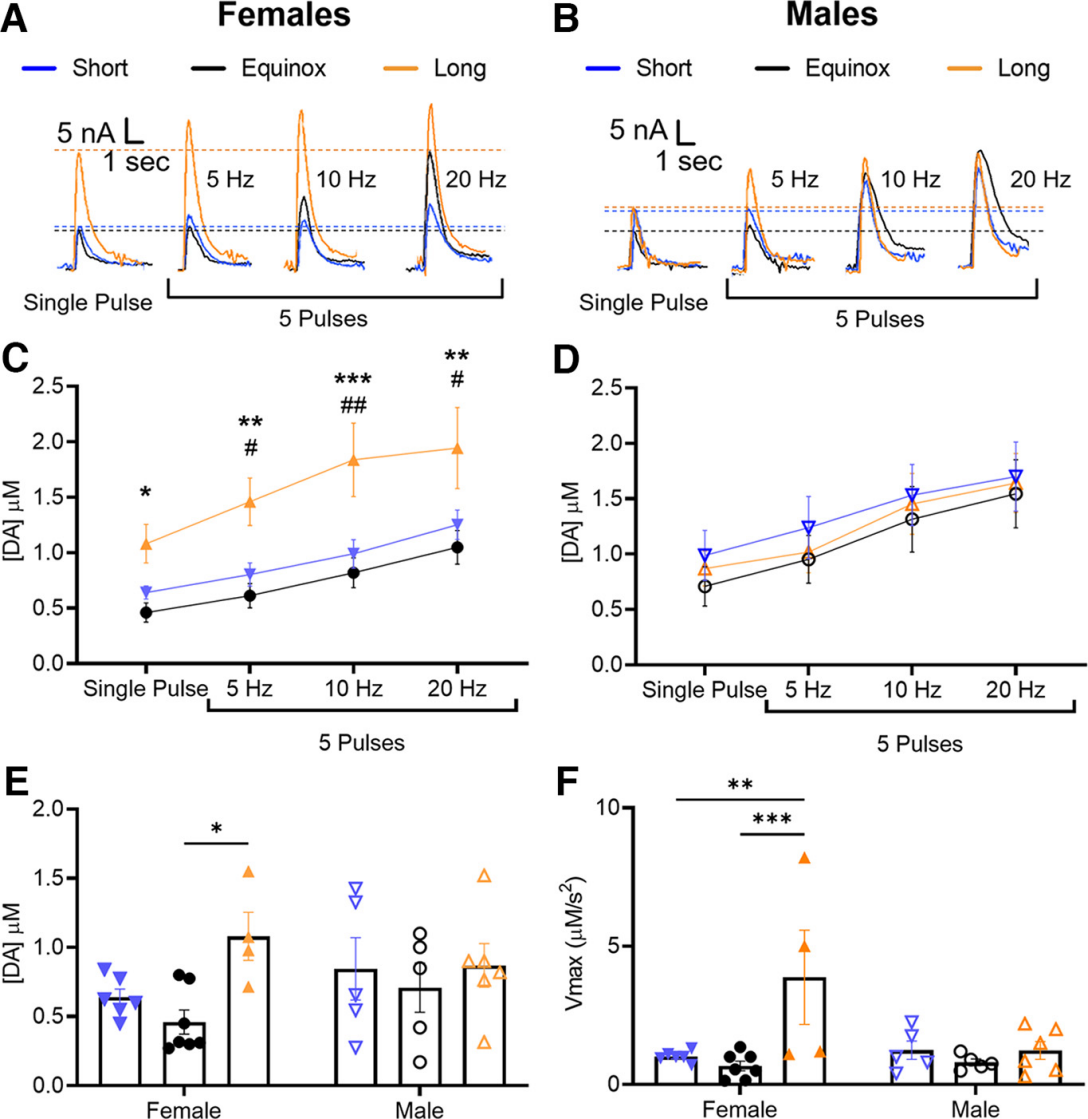
Effects of Long photoperiod on NAc core dopamine release and uptake rate are female-specific. Representative traces from females (***A***) and males (***B***) raised in Short (males *N* = 4, females *N* = 5), Equinox (males *N* = 5, females *N* = 7), and Long (males *N* = 6, females = 4) photoperiods. ***C***, Evoked NAc core dopamine release across tonic and phasic frequencies in females had a significant main effect of photoperiod (*F*_(2,55)_ = 26.69, *p* < 0.0001) and stimulation frequency (*F*_(3,55)_ = 10.26, *p* < 0.0001). ***D***, while males only had a significant main effect of frequency (*F*_(3,48)_ = 5.667, *p* = 0.002). ***E***, Evoked dopamine release from a single electrical pulse in males (open symbols) and females (closed symbols) showed a significant main effect of photoperiod (*F*_(2,27)_ = 3.424, *p* = 0.047). ***F***, Maximal rate of dopamine uptake (*V_max_*) in males and females showed a significant main effect of photoperiod (*F*_(2,27)_ = 6.029, *p* = 0.007) and an interaction effect (*F*_(2,27)_ = 4.334, *p* = 0.023). *p < 0.05, **p < 0.01, ***p < 0.001 for Long versus Equinox; #*p* < 0.05, ##*p* < 0.01 for Long versus Short. Data were analyzed using two-way ANOVA and Tukey’s *post hoc* multiple comparisons test with an α value of *p* = 0.05. Data are represented as mean ± SEM.

As there are known sex differences in the regulation of mesolimbic dopamine (Q.D. Walker et al., 2000; Dluzen and McDermott, 2008; Becker, 2009; Yoest et al., 2014; Calipari et al., 2017; Zachry et al., 2021), we investigated a potential interaction of sex and photoperiod on NAc core dopamine terminal function by analyzing data from male and female mice separately (Fig. 2*A*,*B*). Sample sizes were as follows: Short (males *N* = 4, females *N* = 5), Equinox (males *N* = 5, females *N* = 7), and Long (males *N* = 6, females = 4) where N is number of animals. Using a two-way ANOVA, we found significant effect of stimulation frequency per se in both males (*F*_(3,48)_ = 5.667, *p* = 0.002) and females (*F*_(3,55)_ = 10.26, *p* < 0.0001). Strikingly, we found that Long photoperiod enhanced dopamine release only in females (*F*_(2,55)_ = 26.69, *p* < 0.0001; Fig. 2*C*), while there was no significant effect of photoperiod on NAc core dopamine release in male mice (*F*_(2,48)_ = 0.7842, *p* = 0.4622; Fig. 2*D*). Tukey’s multiple comparison’s *post hoc* tests showed significant differences in NAc core dopamine release between Long and Equinox photoperiod females at the single pulse (*p* = 0.029), 5-Hz (*p* = 0.002), 10-Hz (*p* = 0.0002), and 20-Hz (*p* = 0.001) stimulation frequencies, as well as significant differences between Short and Long photoperiod females at the 5-Hz (*p* = 0.031), 10-Hz (*p* = 0.003), and 20-Hz (*p* = 0.016) stimulation frequencies. Thus, the overall increase in NAc core dopamine release in Long photoperiod described above in Figure 1 reflects sex-specific effects of photoperiod on dopamine release in females. It is also important to note that DA release in NAc core slices from Females in the Long photoperiod group exhibit enhanced dopamine release at all stimulation frequencies tested, indicating that the photoperiod effect we observed is not dependent on stimulation frequency.

To assess possible synaptic mechanisms through which photoperiod exerts its effects on NAc core dopamine, we analyzed concentration of single pulse evoked DA release in females and males (Fig. 2*E*) and for the maximal rate of dopamine uptake (*V_max_*; Fig. 2*F*). Two-way ANOVA revealed a significant main effect of photoperiod on dopamine release (*F*_(2,27)_ = 3.424, *p* = 0.047). Tukey’s *post hoc* multiple comparisons test showed that NAc core from Long photoperiod females had elevated dopamine release compared with those from Equinox photoperiod females (*p* = 0.02). We also found a significant main effect of photoperiod on dopamine uptake (*F*_(2,27)_ = 6.029, *p* = 0.007) as well as a significant interaction of sex and photoperiod (*F*_(2,27)_ = 4.334, *p* = 0.023) using two-way ANOVA. Maximal dopamine uptake in the NAc core was enhanced in Long photoperiod females compared with both Short (*p* = 0.004) and Equinox (*p* = 0.001) photoperiod groups as tested by Tukey’s *post hoc* multiple comparisons test. We did not observe any significant differences in dopamine release or uptake in the males as tested by Tukey’s *post hoc* multiple comparisons test. Taken together, our results indicate photoperiod has female-specific effects on both release and uptake of NAc core dopamine.

### Long photoperiod enhances DAT inhibition in females

**Figure 3.**
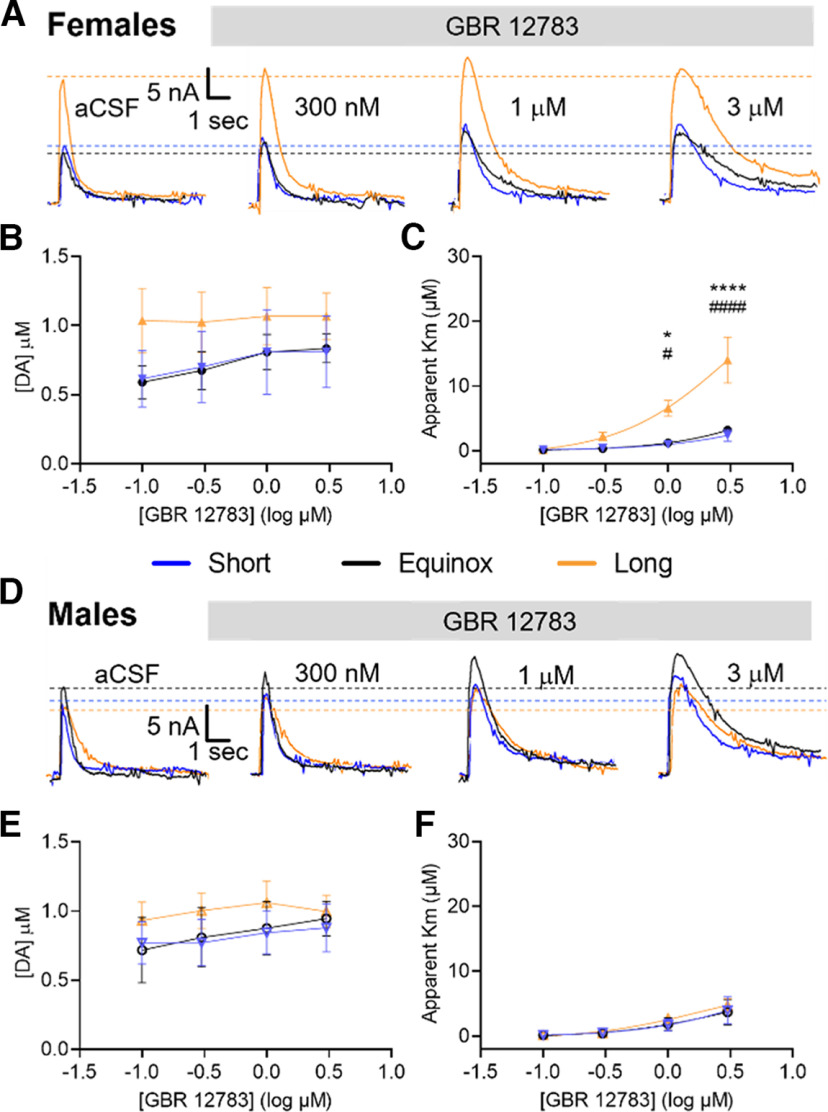
Dopamine release and uptake in the NAc core of Long photoperiod female mice are more sensitive to DAT inhibition by GBR 12783. ***A***, Representative traces of evoked NAc core dopamine release across increasing concentrations of GBR 12783 in Short (males *N* = 4, females *N* = 4), Equinox (males *N* = 4, females *N* = 5), and Long (males *N* = 5, females *N* = 6) photoperiod female mice. Dotted lines denote magnitude of release in control aCSF condition. ***B***, Evoked NAc core dopamine release with increasing concentrations of GBR 12783 had a main effect of photoperiod in females (*F*_(2,44)_ = 3.351, *p* = 0.0442). ***C***, Magnitude of uptake inhibition (apparent *Km*) showed a main effect of photoperiod (*F*_(2,48)_ = 16.21, *p* < 0.0001), GBR 12783 dose (*F*_(3,48)_ = 12.27, *p* < 0.0001), and an interaction effect (*F*_(6,48)_ = 4.585, *p* = 0.0009). ***D***, Representative traces of evoked NAc core dopamine release across increasing concentrations of GBR12783 in Short, Equinox, and Long photoperiod male mice. Dotted lines denote magnitude of release in control aCSF condition. Evoked NAc dopamine release (***E***) and magnitude (***F***) of uptake inhibition with increasing concentrations of GBR 12783 in male mice. *p < 0.05, ****p < 0.0001 for Long versus Equinox; #*p* < 0.05, ####*p* < 0.0001 for Long versus Short. Data were analyzed using two-way ANOVA and Tukey’s *post hoc* multiple comparisons test with an α value of *p* = 0.05. Data are represented as mean ± SEM.

As photoperiod may exert its effects on NAc core dopamine release and uptake in females through modulation of DAT, we examined a role for DAT using a selective DAT inhibitor (GBR 12783). We measured peak dopamine release and uptake and derived changes to apparent DAT *K_m_* across doses of GBR 12783 and photoperiods (Fig. 3). Sample sizes were as follows: Short (males *N* = 4, females *N* = 4), Equinox (males *N* = 4, females *N* = 5), and Long (males *N* = 5, females *N* = 6) where N is number of animals. Consistent with Figure 2*E*, we again confirmed with two-way ANOVA the significant effect of photoperiod on evoked dopamine release in the females (*F*_(2,44)_ = 3.351, *p* = 0.0442; Fig. 3*B*), but not the males (*F*_(2,40)_ = 1.596, *p* = 0.2153; Fig. 3*E*). In addition, in NAc core from female mice the apparent *K_m_* of DAT in response to uptake inhibition showed main effects of photoperiod (*F*_(2,48)_ = 16.21, *p* < 0.0001) and of GBR 12783 dose (*F*_(3,48)_ = 12.27, *p* < 0.0001), as well as a significant interaction of photoperiod and dose (*F*_(6,48)_ = 4.585, *p* = 0.0009; Fig. 3*C*) as tested by two-way ANOVA. We found that the action of GBR 12783 on DAT uptake inhibition was enhanced in slices from Long photoperiod females at 1 μm compared with Short (*p* = 0.019) and Equinox females (*p* = 0.017) and at 3 μm compared with Short (*p* < 0.0001) and Equinox females (*p* < 0.0001) using Tukey’s *post hoc* multiple comparisons test. Thus, pharmacological inhibition of DAT by GBR 12783 was more sensitive in the NAc core of Long photoperiod female mice. We did not find a significant effect of photoperiod on pharmacological DAT inhibition in males (*F*_(2,40)_ = 0.3393, *p* = 0.7143; Fig. 3*F*). Our results showing that GBR 12783 was more potent in Long photoperiod females demonstrates sex-specific photoperiod effects on DAT function in NAc core dopaminergic terminals.

## Discussion

Here, we have laid the groundwork for the broader question of how environmental signals can program or shape the neural circuits that promote affective behavior. We have shown that photoperiod, one of the most pervasive and reliable environmental signals, impacts mesolimbic dopamine signaling, a key component of the neural circuitry that contributes to affective state. Our results, for the first time, demonstrate that photoperiod fine-tunes the subsecond signaling dynamics of neuromodulatory synapses.

Photoperiod-dependent changes in dopamine were only detected in females, where Long summer-like photoperiods enhanced dopamine release and uptake. This female-specific photoperiod effect was present at all stimulation frequencies we tested. This indicates that the enhancement of dopamine release in Long photoperiod females was not dependent on stimulation frequency, suggesting that Long-summer like photoperiod enhances dopamine release in both tonic and phasic firing conditions. There are several possible mechanisms through which this could occur. Cholinergic interneurons (CINs) in the NAc can enhance and even directly elicit dopamine release from terminals in the NAc through presynaptic nicotinic acetylcholine receptors (Yorgason et al., 2017). There is evidence that NAc CIN activity is diurnal and drives diurnal rhythms of NAc dopamine release (Stowe et al., 2022), so it is possible that CIN firing rate may be responsive to photoperiod. Another possible mechanism through which photoperiod could affect dopamine release is through regulation of D2 dopamine autoreceptors. Previous work has shown that the ratio of D2 to D1 dopamine receptor expression in the striatum shifts to increase D2 and decrease D1 expression in a mutant clock gene mouse model (Spencer et al., 2012), which warrants further investigation as to whether photoperiod may also regulate the D2 receptor to exert its effects on dopamine release.

We also found that dopamine uptake was enhanced in the Long-photoperiod females. Because DAT activity can influence dopamine release through the recycling of transmitter (Egaña et al., 2009; Condon et al., 2019), it is also possible that the enhanced DAT activity in Long photoperiod females also enhances dopamine release. Using the DAT inhibitor, GBR 12783, we found that Long photoperiod modulated the sensitivity of DAT to pharmacological inhibition in females, consistent with our findings that female baseline DAT activity is also different by photoperiod. If photoperiod’s effect on baseline DAT function drives the differences we see in dopamine release, one would expect that inhibition of DAT should decrease dopamine release. However, we found that inhibiting DAT using GBR 12783 did not have a significant effect on dopamine release at any of the concentrations we tested. It has been shown that GBR 12783 actually enhances dopamine release in striatal synaptosomes (Bonnet and Costentin, 1986), potentially occluding any decreases in dopamine release. Taken together, further studies are necessary to determine the mechanism through which dopamine release is modulated by photoperiod.

Previous studies have shown that increased DAT expression in genetic and experience-dependent models in rodents does not affect the potency of DAT inhibitors (Calipari et al., 2013, 2014). Rather, functional modifications to DAT, such as phosphorylation at threonine 53, enhance baseline dopamine uptake and potency of DAT inhibitors (Morón et al., 2003; Foster et al., 2012; Calipari et al., 2017). Thus, because we found photoperiod-dependent differences in DAT inhibitor potency in females, we suggest it is likely that photoperiod acts on dopamine signaling at least in part through increased DAT function through regulation of DAT phosphorylation.

The sex-specific effects of photoperiod we have observed here warrant further investigation of the mechanisms through which photoperiod affects males and females differently. There are at least three major biological phenomena that can underlie sexually dimorphic phenotypes: sex hormone action in the adult organism, sex hormone action during development, and genetic influence of sex chromosomes (Becker et al., 2005; McCarthy and Arnold, 2011). Estrous cycle-dependent and sex-hormone regulation of the adult mesolimbic dopamine system is well established (Becker, 2009; Yoest et al., 2014; Calipari et al., 2017; Becker and Chartoff, 2019), and decades of literature have examined how photoperiod affects sex hormone regulation with respect to seasonal mating behaviors (Ortavant et al., 1988; Luboshitzky and Lavie, 1999; Walton et al., 2011; Borniger and Nelson, 2017). Furthermore, studies have shown a link between photoperiod, melatonin, and sex hormones in humans (Reiter, 1998; Luboshitzky and Lavie, 1999; Kuzmenko et al., 2021). It is possible that photoperiod influences the estrous-cycle to exert female-specific effects on synaptic dopamine dynamics, as previous work has shown that females in estrous show enhanced dopamine release and uptake (Calipari et al., 2017) similar to the females raised in Long photoperiod. Photoperiod has also been linked to regulation of puberty onset and sex hormones during the peripubertal period (Ebling and Foster, 1989; Adam and Robinson, 1994; Ebling, 2010; Bohlen et al., 2018), which is a critical developmental period that affects the mesolimbic dopamine system (Becker, 2009; Kuhn et al., 2010; D.M. Walker et al., 2017) as well as reward-related behaviors (Yoest et al., 2014; Harden et al., 2018; Becker and Chartoff, 2019). Future research using multiple developmental timepoints may reveal whether female-specific photoperiod modulation of dopamine signaling depends on a critical period of development. To our knowledge, studies have not examined how sex chromosomes influence photoperiodism in mammals. However, sex chromosomes, independent of gonadal phenotype, have been directly implicated in sex differences in the dopamine system (Carruth et al., 2002; Dewing et al., 2006), and have even been shown to play a role in regulating reward-related behavior (Barker et al., 2010). Future studies using the four core genotype model should examine the mechanisms through which sex-specific effects of photoperiod could emerge across development either by sex chromosomes or through gonadal hormones.

We were intrigued to find that dopamine release and uptake in the NAc core of male mice were not significantly influenced by photoperiod, especially since previous work using males have shown photoperiod can impact the striatal dopamine system by several measures. Findings from Goda et al. (2015) showed that longer photoperiod increased tissue dopamine content in the striatum of male chipmunks. Additionally, dopamine turnover was blunted in the NAc of male mice raised in winter-like short photoperiod (Itzhacki et al., 2018). In humans, striatal presynaptic dopamine synthesis was found to be enhanced in fall/winter months in both men and women (Eisenberg et al., 2010). Extracellular dopamine concentration has been shown to have an inverse relationship with evoked synaptic dopamine release (Ferris et al., 2014), and dopamine synthesis rate can paradoxically change in the opposite direction from overall dopamine tissue content (Gainetdinov et al., 1998). As previously mentioned, our study is the first to examine photoperiod’s effect on the functional subsecond signaling of dopamine in the NAc core. As such, it is possible that photoperiod may indeed affect measures of dopamine in males as assessed by the aforementioned studies independently from our measures of synaptic dopamine dynamics. Further studies are needed to understand the effect of photoperiod on the male dopamine system and how it may differ from females to produce seasonal changes in behavior.

Another important distinction between previous photoperiod studies and our own is our use of the melatonin-competent C3Hf^+/+^ strain. The commonly used C57/BL6J strain (C57), does not produce physiological levels of endogenous melatonin because of genetic mutations in the melatonin synthesis pathway (Ebihara et al., 1986; Goto et al., 1989). Melatonin is a hormone that is released from the pineal gland at the onset of darkness and is inhibited by the onset of light, effectively encoding photoperiod. Therefore, melatonin is considered a main biological signal through which seasonal light information is transduced (Brzezinski, 1997; Hardeland et al., 2006). Interestingly, Green et al. (2015) showed that photoperiodic influence of affective behaviors in C3Hf^+/+^ mice depends on melatonin signaling through the melatonin MT1 receptor (Green et al., 2015). The species-specific effect of photoperiod on striatal dopamine content reported by Goda et al. (2015) could therefore be explained by the fact that chipmunks endogenously produce melatonin while C57 mice do not. The only commonly used lab strain of mice that produce endogenous melatonin is the C3H strain (Goto et al., 1989). This strain is homozygous for a mutation in the *rd* gene that results in rod/cone degeneration rendering them visually impaired (Tosini and Menaker, 1998), but as they retain their circadian photosensitive ganglion cells, they still entrain to circadian photoperiods (Panda et al., 2003). The C3Hf^+/+^ strain has had the retinal degeneration mutation bred out (Contreras-Alcantara et al., 2012) so that mice retain normal vision for behavioral experiments, are sensitive to photoperiodic light information, and are melatonin competent.

Several studies have shown that nearly all aspects of dopamine transmission, including synaptic dopamine release, DAT activity, expression of dopamine receptors, dopamine synthesis and dopamine-dependent behaviors show diurnal rhythms (Naber et al., 1980; Schade et al., 1995; Akhisaroglu et al., 2005; Sleipness et al., 2007; Ferris et al., 2014; Stowe et al., 2022). Experiments in the present study were conducted during the mid-light phase of each photoperiod where synaptic dopamine release and DAT function are predicted to be at their peak (Ferris et al., 2014). Using a single time point, we cannot determine whether photoperiod changes the amplitude, period, or waveform of the known diurnal rhythm of NAc dopamine dynamics to result in the differences we report here, and additional time point studies around the clock will be needed.

Previous work from our group used the same mouse model and photoperiod conditions to test how seasonally relevant photoperiods impact affective behaviors (Green et al., 2015). Mice raised in the Long summer-like photoperiod showed less anxiodepressive-like behaviors than mice raised in Short or Equinox photoperiods, as measured by forced swim test and elevated zero maze (Green et al., 2015). The effect of photoperiod on synaptic dopamine dynamics that we report here may influence dopamine-dependent affective behaviors beyond the anxiodepressive behaviors examined by Green et al. (2015). NAc core dopamine is highly involved in reinforcement learning (Tsai et al., 2009; Floresco, 2015; Kutlu et al., 2021), and our data predicts that photoperiod could drive differences in female reinforcement behaviors such as reward responding, acquisition, and motivation through modulation of NAc core dopamine. Stowe et al., 2022 demonstrated that the diurnal rhythm of sign-tracking behavior is synchronous with diurnal rhythms of phasic/tonic ratio of electrically evoked NAc core dopamine. Sign-tracking is an associative learning behavior dependent on NAc dopamine that is linked to addiction-related behaviors (Robinson et al., 2014). Based on our findings, we would predict that females raised in Long photoperiod would exhibit more sign-tracking behavior than males or females raised in other photoperiods. Such studies would be especially pertinent as evidence suggests that substance use disorders show seasonality as well (Sandyk and Kanofsky, 1992; Sher, 2004; Morales-Muñoz et al., 2017; Barbosa-Méndez and Salazar-Juárez, 2020).

Cocaine-related behaviors in rodents have also been shown to be circadian (Baird and Gauvin, 2000; Akhisaroglu et al., 2004; McClung, 2007; Falcón and McClung, 2009; Parekh and McClung, 2016; Depoy et al., 2017; Depoy et al., 2021), and disrupting circadian rhythm via mutations in the clock gene, *Npas2*, has female-specific effects on vulnerability to substance use (Depoy et al., 2017; Depoy et al., 2021). *Npas2* mutant females, especially in the dark phase, showed increased cocaine self-administration, faster acquisition of cocaine self-administration, higher break point ratio, and increased extinction responding (Depoy et al., 2017; Depoy et al., 2021). These effects were abolished with ovariectomy of *Npas2* mutant females, suggesting that ovarian hormones mediate these female-specific, circadian-dependent effects (Depoy et al., 2017; Depoy et al., 2021). Because they have more robust dopamine signaling, it is possible that females raised in Long photoperiods may be more vulnerable to increased substance use in response to circadian disruption. In fact, photoperiod has been shown to impact cocaine reinstatement behaviors, where switching to shorter photoperiods decreased cocaine-induced reinstatement, while switching to longer photoperiods had no significant effects (Sorg et al., 2011). However, this study used exclusively male rats, so future studies using females may reveal differential effects of photoperiod on cocaine reinstatement and other substance use behaviors.

The mesolimbic dopamine system is implicated in the pathophysiology of many neuropsychiatric disorders, including major depressive disorder (MDD; Nestler and Carlezon, 2006; Øverland et al., 2019), seasonal affective disorder (SAD; Rosen et al., 1990; Praschak-Rieder and Willeit, 2012), and bipolar disorder (BD; Cousins et al., 2009). Multiple studies have shown that seasonality of depressive symptoms in MDD is more prevalent in women (Otte et al., 2016), and that there is a higher prevalence of SAD in women than in men (Magnusson, 2000). Additionally, more women exhibit seasonality in their BD symptoms than men with BD (Arnold, 2003; Geoffroy et al., 2014). The female sex-specific effects of photoperiod on NAc dopamine dynamics shown here, along with evidence of female-specific circadian effects on dopamine-dependent behaviors (Depoy et al., 2017; Depoy et al., 2021), indicate the possibility that the female dopamine system is more sensitive to circadian light information than males. Thus, females may experience more fluctuation or seasonal changes to the dopamine system contributing to female-specific seasonality of certain neuropsychiatric disorders. These data open the door to future investigation of the mechanisms of sex differences in seasonality of neuropsychiatric disorders.

Taken together, our study demonstrates that photoperiod impacts the subsecond release and uptake dynamics of synaptic mesolimbic dopamine, revealing a novel process by which photoperiod impacts neural systems involved in reward and affective behaviors. These findings identify a potential role for the dopamine system in mediating sex differences in seasonality on a synaptic basis, and an animal model in which to further explore potential mechanisms of sex differences in neuropsychiatric disorders.

## Synthesis

Reviewing Editor: Tod E Kippin, University of California Berkeley Decisions are customarily a result of the Reviewing Editor and the peer reviewers coming together and discussing their recommendations until a consensus is reached. When revisions are invited, a fact-based synthesis statement explaining their decision and outlining what is needed to prepare a revision will be listed below. The following reviewer(s) agreed to reveal their identity: Mark Ferris. Note: If this manuscript was transferred from JNeurosci and a decision was made to accept the manuscript without peer review, a brief statement to this effect will instead be what is listed below.

I have received two independent reviews and both feel the manuscript is acceptable but will two very minor edits.

The remaining issues are:

The paragraph at line 394 in the discussion should be broken up into 2 paragraphs

In addition, Page 14 in the paper (page 15 on the pdf) is blank (not sure if this was intentional)

## References

[B1] Adam CL, Robinson JJ (1994) The role of nutrition and photoperiod in the timing of puberty. Proc Nutr Soc 53:89–102. 10.1079/pns19940013 7913222

[B2] Akhisaroglu M, Ahmed R, Kurtuncu M, Manev H, Uz T (2004) Diurnal rhythms in cocaine sensitization and in Period1 levels are common across rodent species. Pharmacol Biochem Behav 79:37–42. 10.1016/j.pbb.2004.06.014 15388282

[B3] Akhisaroglu M, Kurtuncu M, Manev H, Uz T (2005) Diurnal rhythms in quinpirole-induced locomotor behaviors and striatal D2/D3 receptor levels in mice. Pharmacol Biochem Behav 80:371–377. 10.1016/j.pbb.2004.11.016 15740778

[B4] Arnold LM (2003) Gender differences in bipolar disorder. Psychiatr Clin North Am 26:595–620. 10.1016/s0193-953x(03)00036-4 14563100

[B5] Baird T, Gauvin D (2000) Characterization of cocaine self-administration and pharmacokinetics as a function of time of day in the rat. Pharmacol Biochem Behav 65:289–299. 10.1016/S0091-3057(99)00207-5 10672982

[B6] Barbosa-Méndez S, Salazar-Juárez A (2020) Melatonin decreases cocaine-induced locomotor activity in pinealectomized rats. Braz J Psychiatry 42:295–308. 10.1590/1516-4446-2018-0400 31859790 PMC7236171

[B7] Barker JM, Torregrossa MM, Arnold AP, Taylor JR (2010) Dissociation of genetic and hormonal influences on sex differences in alcoholism-related behaviors. J Neurosci 30:9140–9144. 10.1523/JNEUROSCI.0548-10.2010 20610747 PMC2921163

[B8] Becker JB (2009) Sexual differentiation of motivation: a novel mechanism? Horm Behav 55:646–654. 10.1016/j.yhbeh.2009.03.014 19446081 PMC2684520

[B9] Becker JB, Chartoff E (2019) Sex differences in neural mechanisms mediating reward and addiction. Neuropsychopharmacology 44:166–183. 10.1038/s41386-018-0125-6 29946108 PMC6235836

[B10] Becker JB, Arnold AP, Berkley KJ, Blaustein JD, Eckel LA, Hampson E, Herman JP, Marts S, Sadee W, Steiner M, Taylor J, Young E (2005) Strategies and methods for research on sex differences in brain and behavior. Endocrinology 146:1650–1673. 10.1210/en.2004-1142 15618360

[B11] Bohlen TM, Silveira MA, Buonfiglio DC, Ferreira-Neto HC, Cipolla-Neto J, Donato J, Frazao R (2018) A short-day photoperiod delays the timing of puberty in female mice via changes in the kisspeptin system. Front Endocrinol 9:44. 29515520 10.3389/fendo.2018.00044 PMC5826198

[B12] Bonnet JJ, Costentin J (1986) GBR 12783, a potent and selective inhibitor of dopamine uptake: biochemical studies in vivo and ex vivo. Eur J Pharmacol 121:199–209. 10.1016/0014-2999(86)90491-7 3754516

[B13] Borniger JC, Nelson RJ (2017) Photoperiodic regulation of behavior: peromyscus as a model system. Semin Cell Dev Biol 61:82–91. 10.1016/j.semcdb.2016.06.015 27346738

[B14] Brzezinski A (1997) Melatonin in humans. N Engl J Med 336:186–195. 10.1056/NEJM199701163360306 8988899

[B15] Calipari ES, Ferris MJ, Salahpour A, Caron MG, Jones SR (2013) Methylphenidate amplifies the potency and reinforcing effects of amphetamines by increasing dopamine transporter expression. Nat Commun 4:2720. 10.1038/ncomms3720 24193139 PMC4017736

[B16] Calipari ES, Ferris MJ, Melchior JR, Bermejo K, Salahpour A, Roberts DCS, Jones SR (2014) Methylphenidate and cocaine self-administration produce distinct dopamine terminal alterations. Addict Biol 19:145–155. 10.1111/j.1369-1600.2012.00456.x 22458761 PMC3390453

[B17] Calipari ES, Juarez B, Morel C, Walker DM, Cahill ME, Ribeiro E, Roman-Ortiz C, Ramakrishnan C, Deisseroth K, Han MH, Nestler EJ (2017) Dopaminergic dynamics underlying sex-specific cocaine reward. Nat Commun 8:13877. 10.1038/ncomms13877 28072417 PMC5234081

[B18] Carlsson A, Svennerholm L, Winblad B (1980) Seasonal and circadian monoamine variations in human brains examined post mortem. Acta Psychiatr Scand 61:75–85. 10.1111/acps.1980.61.s280.75 28678362

[B19] Carruth LL, Reisert I, Arnold AP (2002) Sex chromosome genes directly affect brain sexual differentiation. Nat Neurosci 5:933–934. 10.1038/nn922 12244322

[B20] Cohen JY, Haesler S, Vong L, Lowell BB, Uchida N (2012) Neuron-type-specific signals for reward and punishment in the ventral tegmental area. Nature 482:85–88. 10.1038/nature10754 22258508 PMC3271183

[B21] Condon MD, Platt NJ, Zhang Y-F, Roberts BM, Clements MA, Vietti-Michelina S, Tseu M-Y, Brimblecombe KR, Threlfell S, Mann EO, Cragg SJ (2019) Plasticity in striatal dopamine release is governed by release-independent depression and the dopamine transporter. Nat Commun 10:4263. 10.1038/s41467-019-12264-9 31537790 PMC6753151

[B22] Contreras-Alcantara S, Baba K, Tosini G (2012) Removal of melatonin receptor type 1 induces insulin resistance in the mouse. Obesity (Silver Spring) 18:1861–1863. 10.1038/oby.2010.24 PMC2929321 20168308

[B23] Cousins D, Butts K, Young A (2009) The role of dopamine in bipolar disorder. Bipolar Disord 11:787–806. 10.1111/j.1399-5618.2009.00760.x 19922550

[B24] Depoy LM, McClung CA, Logan RW (2017) Neural mechanisms of circadian regulation of natural and drug reward. Neural Plast 2017:5720842–14. 10.1155/2017/5720842 29359051 PMC5735684

[B25] Depoy LM, Becker-Krail DD, Zong W, Petersen K, Shah NM, Brandon JH, Miguelino AM, Tseng GC, Logan RW, McClung CA (2021) Circadian-dependent and sex-dependent increases in intravenous cocaine self-administration in Npas2 mutant mice. J Neurosci 41:1046–1058. 10.1523/JNEUROSCI.1830-20.2020 33268545 PMC7880289

[B26] Dewing P, Chiang CWK, Sinchak K, Sim H, Fernagut PO, Kelly S, Chesselet MF, Micevych PE, Albrecht KH, Harley VR, Vilain E (2006) Direct regulation of adult brain function by the male-specific factor SRY. Curr Biol 16:415–420. 10.1016/j.cub.2006.01.017 16488877

[B27] Dluzen DE, McDermott JL (2008) Sex differences in dopamine- and vesicular monoamine-transporter functions: implications for methamphetamine use and neurotoxicity. Ann N Y Acad Sci 1139:140–150. 10.1196/annals.1432.010 18991858

[B28] Ebihara S, Marks T, Hudson DJ, Menaker M (1986) Genetic control of melatonin synthesis in the pineal gland of the mouse. Science 231:491–493. 10.1126/science.3941912 3941912

[B29] Ebling FJP (2010) Photoperiodic regulation of puberty in seasonal species. Mol Cell Endocrinol 324:95–101. 10.1016/j.mce.2010.03.018 20347928

[B30] Ebling F, Foster D (1989) Pineal melatonin rhythms and the timing of puberty in mammals. Experientia 45:946–954. 10.1007/BF01953052 2680575

[B31] Egaña LA, Cuevas RA, Baust TB, Parra LA, Leak RK, Hochendoner S, Peña K, Quiroz M, Hong WC, Dorostkar MM, Janz R, Sitte HH, Torres GE (2009) Physical and functional interaction between the dopamine transporter and the synaptic vesicle protein synaptogyrin-3. J Neurosci 29:4592–4604. 10.1523/JNEUROSCI.4559-08.2009 19357284 PMC2846176

[B32] Eisenberg DP, Kohn PD, Baller EB, Bronstein JA, Masdeu JC, Berman KF (2010) Seasonal effects on human striatal presynaptic dopamine synthesis. J Neurosci 30:14691–14694. 10.1523/JNEUROSCI.1953-10.2010 21048126 PMC3010858

[B33] Erickson JD, Eiden LE, Hoffman BJ (1992) Expression cloning of a reserpine-sensitive vesicular monoamine transporter. Proc Natl Acad Sci U S A 89:10993–10997. 10.1073/pnas.89.22.10993 1438304 PMC50469

[B34] Falcón E, McClung CA (2009) A role for the circadian genes in drug addiction. Neuropharmacology 56:91–96. 10.1016/j.neuropharm.2008.06.054 18644396 PMC2635341

[B35] Ferris MJ, España RA, Locke JL, Konstantopoulos JK, Rose JH, Chen R, Jones SR (2014) Dopamine transporters govern diurnal variation in extracellular dopamine tone. Proc Natl Acad Sci U S A 111:2751–2759. 10.1073/pnas.1407935111 PMC4084435 24979798

[B36] Floresco SB (2015) The nucleus accumbens: an interface between cognition, emotion, and action. Annu Rev Psychol 66:25–52. 10.1146/annurev-psych-010213-115159 25251489

[B37] Foster JD, Yang JW, Moritz AE, ChallaSivaKanaka S, Smith MA, Holy M, Wilebski K, Sitte HH, Vaughan RA (2012) Dopamine transporter phosphorylation site threonine 53 regulates substrate reuptake and amphetamine-stimulated efflux. J Biol Chem 287:29702–29712. 10.1074/jbc.M112.367706 22722938 PMC3436161

[B38] Gainetdinov RR, Jones SR, Fumagalli F, Wightman RM, Caron MG (1998) Re-evaluation of the role of the dopamine transporter in dopamine system homeostasis. Brain Res Brain Res Rev 26:148–153. 10.1016/s0165-0173(97)00063-5 9651511

[B39] Geoffroy PA, Bellivier F, Scott J, Etain B (2014) Seasonality and bipolar disorder: a systematic review, from admission rates to seasonality of symptoms. J Affect Disord 168:210–223. 10.1016/j.jad.2014.07.002 25063960

[B40] Giros B, Jaber M, Jones SR, Wightman RM, Caron MG (1996) Hyperlocomotion and indifference to cocaine and amphetamine in mice lacking the dopamine transporter. Nature 379:606–612. 10.1038/379606a0 8628395

[B41] Goda R, Otsuka T, Iwamoto A, Kawai M, Shibata S, Furuse M, Yasuo S (2015) Serotonin levels in the dorsal raphe nuclei of both chipmunks and mice are enhanced by long photoperiod, but brain dopamine level response to photoperiod is species-specific. Neurosci Lett 593:95–100. 10.1016/j.neulet.2015.03.035 25797183

[B42] Goto M, Oshima I, Tomita T, Ebihara S (1989) Melatonin content of the pineal gland in different mouse strains. J Pineal Res 7:195–204. 10.1111/j.1600-079x.1989.tb00667.x 2769571

[B43] Green NH, Jackson CR, Iwamoto H, Tackenberg MC, McMahon DG (2015) Photoperiod programs dorsal raphe serotonergic neurons and affective behaviors. Curr Biol 25:1389–1394. 10.1016/j.cub.2015.03.050 25959961 PMC4445239

[B44] Hardeland R, Pandi-Perumal SR, Cardinali DP (2006) Melatonin. Int J Biochem Cell Biol 38:313–316. 10.1016/j.biocel.2005.08.020 16219483

[B45] Harden KP, Mann FD, Grotzinger AD, Patterson MW, Steinberg L, Tackett JL, Tucker-Drob EM (2018) Developmental differences in reward sensitivity and sensation seeking in adolescence: testing sex-specific associations with gonadal hormones and pubertal development. J Pers Soc Psychol 115:161–178. 10.1037/pspp0000172 29094961 PMC5932293

[B46] Itzhacki J, Clesse D, Goumon Y, van Someren EJ, Mendoza J (2018) Light rescues circadian behavior and brain dopamine abnormalities in diurnal rodents exposed to a winter-like photoperiod. Brain Struct Funct 223:2641–2652. 10.1007/s00429-018-1655-8 29560509

[B47] Jones SR, Gainetdinov RR, Mark Wightman R, Caron MG (1998) Mechanisms of amphetamine action revealed in mice lacking the dopamine transporter. J Neurosci 18:1979–1986. 10.1523/JNEUROSCI.18-06-01979.1998 9482784 PMC6792915

[B48] Kim DR, Czarkowski KA, Epperson CN (2011) The relationship between bipolar disorder, seasonality, and premenstrual symptoms. Curr Psychiatry Rep 13:500–503. 10.1007/s11920-011-0233-z 21918807 PMC4107419

[B49] Kuhn C, Johnson M, Thomae A, Luo B, Simon SA, Zhou G, Walker QD (2010) The emergence of gonadal hormone influences on dopaminergic function during puberty. Horm Behav 58:122–137. 10.1016/j.yhbeh.2009.10.015 19900453 PMC2883625

[B50] Kutlu MG, Zachry JE, Melugin PR, Cajigas SA, Chevee MF, Kelly SJ, Kutlu B, Tian L, Siciliano CA, Calipari ES (2021) Dopamine release in the nucleus accumbens core signals perceived saliency. Curr Biol 31:4748–4761.e8. 10.1016/j.cub.2021.08.052 34529938 PMC9084920

[B51] Kuzmenko N. v, Tsyrlin VA, Pliss MG (2021) Seasonal dynamics of melatonin, prolactin, sex hormones, and adrenal hormones in healthy people: a meta-analysis. J Evol Biochem Phys 57:451–472. 10.1134/S0022093021030029

[B52] Luboshitzky R, Lavie P (1999) Melatonin and sex hormone interrelationships-a review. J Pediatr Endocrinol Metab 12:355–362. 10.1515/jpem.1999.12.3.355 10821215

[B53] Ljungberg T, Apicella P, Schultz W (1992) Responses of monkey dopamine neurons during learning of behavioral reactions. J Neurophysiol 67:145–163. 10.1152/jn.1992.67.1.145 1552316

[B54] Magnusson A (2000) An overview of epidemiological studies on seasonal affective disorder. Acta Psychiatr Scand 101:176–184. 10.1046/j.0902-4441.2000.x 10721866

[B55] McCarthy MM, Arnold AP (2011) Reframing sexual differentiation of the brain. Nat Neurosci 14:677–683. 10.1038/nn.2834 21613996 PMC3165173

[B56] McClung C (2007) Circadian rhythms, the mesolimbic dopaminergic circuit, and drug addiction. ScientificWorldJournal 7:194–202. 10.1100/tsw.2007.213 17982593 PMC5901358

[B57] Melrose S (2015) Seasonal affective disorder: an overview of assessment and treatment approaches. Depress Res Treat 2015:178564–178566. 10.1155/2015/178564 26688752 PMC4673349

[B58] Mogenson GJ, Jones DL, Yim CY (1980) From motivation to action: functional interface between the limbic system and the motor system. Prog Neurobiol 14:69–97. 10.1016/0301-0082(80)90018-0 6999537

[B59] Morales-Muñoz I, Koskinen S, Partonen T (2017) Seasonal affective disorder and alcohol abuse disorder in a population-based study. Psychiatry Res 253:91–98. 10.1016/j.psychres.2017.03.029 28364591

[B60] Morón JA, Zakharova I, Ferrer J. v, Merrill GA, Hope B, Lafer EM, Lin ZC, Wang JB, Javitch JA, Galli A, Shippenberg TS (2003) Mitogen-activated protein kinase regulates dopamine transporter surface expression and dopamine transport capacity. J Neurosci 23:8480–8488. 10.1523/JNEUROSCI.23-24-08480.2003 13679416 PMC6740378

[B61] Naber D, Wirz-Justice A, Kafka M, Wehr T (1980) Dopamine receptor binding in rat striatum: ultradian rhythm and its modification by chronic imipramine. Psychopharmacology (Berl) 68:1–5. 10.1007/BF00426642 6771793

[B62] Nestler EJ, Carlezon WA (2006) The mesolimbic dopamine reward circuit in depression. Biol Psychiatry 59:1151–1159. 10.1016/j.biopsych.2005.09.018 16566899

[B63] Neumeister A, Willeit M, Praschak-Rieder N, Asenbaum S, Stastny J, Hilger E, Pirker W, Konstantinidis A, Kasper S (2001) Dopamine transporter availability in symptomatic depressed patients with seasonal affective disorder and healthy controls. Psychol Med 31:1467–1473. 10.1017/s003329170105434z 11722161

[B64] Nolan SO, Zachry JE, Johnson AR, Brady LJ, Siciliano CA, Calipari ES (2020) Direct dopamine terminal regulation by local striatal microcircuitry. J Neurochem 155:475–493. 10.1111/jnc.15034 32356315 PMC7606645

[B65] Ortavant R, Bocquier F, Pelletier J, Ravault JP, Thimonier J, Volland-Nail P (1988) Seasonality of reproduction in sheep and its control by photoperiod. Aust J Biol Sci 41:69–85. 3077741

[B66] Otte C, Gold SM, Penninx BW, Pariante CM, Etkin A, Fava M, Mohr DC, Schatzberg AF (2016) Major depressive disorder. Nat Rev Dis Primers 2:16065. 10.1038/nrdp.2016.65 27629598

[B67] Øverland S, Woicik W, Sikora L, Whittaker K, Heli H, Skjelkvåle FS, Sivertsen B, Colman I (2019) Seasonality and symptoms of depression: a systematic review of the literature. Epidemiol Psychiatr Sci 29:e31. 10.1017/S2045796019000209 31006406 PMC8061295

[B680] Panda S, Provencio I, Tu DC, Pires SS, Rollag MD, Castrucci AM, Pletcher MT, Sato TK, Wiltshire T, Andahazy M, Kay SA, Van Gelder RN, Hogenesch JB (2003) Melanopsin is required for non-image-forming photic responses in blind mice. Science 301:525–527. 12829787 10.1126/science.1086179

[B68] Parekh PK, McClung CA (2016) Circadian mechanisms underlying reward-related neurophysiology and synaptic plasticity. Front Psychiatry 6:1–11. 10.3389/fpsyt.2015.00187 PMC4709415 26793129

[B69] Praschak-Rieder N, Willeit M (2012) Imaging of seasonal affective disorder and seasonality effects on serotonin and dopamine function in the human brain. Curr Top Behav Neurosci 11:149–167. 10.1007/7854_2011_174 22218931

[B70] Reiter RJ (1998) Melatonin and human reproduction. Ann Med 30:103–108. 10.3109/07853899808999391 9556096

[B71] Robinson T, Yager L, Cogan E, Saunders B (2014) On the motivational properties of reward cues: individual differences. Neuropharmacology 76:450–459. 10.1016/j.neuropharm.2013.05.040 23748094 PMC3796005

[B72] Rosen LN, Targum SD, Terman M, Bryant MJ, Hoffman H, Kasper SF, Hamovit JR, Docherty JP, Welch B, Rosenthal NE (1990) Prevalence of seasonal affective disorder at four latitudes. Psychiatry Res 31:131–144. 10.1016/0165-1781(90)90116-m 2326393

[B73] Sandyk R, Kanofsky JD (1992) Cocaine addiction: relationship to seasonal affective disorder. Int J Neurosci 64:195–201. 10.3109/00207459209000545 1342039

[B74] Schade R, Vick K, Ott T, Sohr R, Pfister C, Bellach J, Golor G, Lemmer B (1995) Circadian rhythms of dopamine and cholecystokinin in nucleus accumbens and striatum of rats – influence on dopaminergic stimulation. Chronobiol Int 12:87–99. 10.3109/07420529509064504 8653803

[B75] Schultz W (1998) The phasic reward signal of primate dopamine neurons. Adv Pharmacol 42:686–690. 10.1016/s1054-3589(08)60841-8 9327992

[B76] Schultz W (2007) Multiple dopamine functions at different time courses. Annu Rev Neurosci 30:259–288. 10.1146/annurev.neuro.28.061604.135722 17600522

[B77] Sher L (2004) Alcoholism and seasonal affective disorder. Compr Psychiatry 45:51–56. 10.1016/j.comppsych.2003.09.007 14671737

[B78] Shin JH, Adrover MF, Alvarez VA (2017) Distinctive modulation of dopamine release in the nucleus accumbens shell mediated by dopamine and acetylcholine receptors. J Neurosci 37:11166–11180. 10.1523/JNEUROSCI.0596-17.2017 29030431 PMC5688525

[B79] Siemann JK, Grueter BA, McMahon DG (2021) Rhythms, reward, and blues: consequences of circadian photoperiod on affective and reward circuit function. Neuroscience 457:220–234. 10.1016/j.neuroscience.2020.12.010 33385488 PMC7897275

[B80] Sleipness E, Sorg B, Jansen H (2007) Diurnal differences in dopamine transporter and tyrosine hydroxylase levels in rat brain: dependence on the suprachiasmatic nucleus. Brain Res 1129:34–42. 10.1016/j.brainres.2006.10.063 17156761

[B81] Sørensen G, Rickhag M, Leo D, Lycas MD, Ridderstrøm PH, Weikop P, Lilja JH, Rifes P, Herborg F, Woldbye D, Wörtwein G, Gainetdinov RR, Fink-Jensen A, Gether U (2021) Disruption of the PDZ domain-binding motif of the dopamine transporter uniquely alters nanoscale distribution, dopamine homeostasis, and reward motivation. J Biol Chem 297:1–17. 10.1016/j.jbc.2021.101361 PMC8648841 34756883

[B82] Sorg BA, Stark G, Sergeeva A, Jansen HT (2011) Photoperiodic suppression of drug reinstatement. Neuroscience 176:284–295. 10.1016/j.neuroscience.2010.12.022 21185915 PMC3040258

[B83] Spencer S, Torres-Altoro MI, Falcon E, Arey R, Marvin M, Goldberg M, Bibb JA, McClung CA (2012) A mutation in CLOCK leads to altered dopamine receptor function. J Neurochem 123:124–134. 10.1111/j.1471-4159.2012.07857.x 22757753 PMC3438377

[B84] Stowe TA, Pitts EG, Leach AC, Iacino MC, Niere F, Graul B, Raab-Graham KF, Yorgason JT, Ferris MJ (2022) Diurnal rhythms in cholinergic modulation of rapid dopamine signals and associative learning in the striatum. Cell Rep 39:110633. 10.1016/j.celrep.2022.110633 35385720 PMC9148619

[B85] Sulzer D, Cragg SJ, Rice ME (2016) Striatal dopamine neurotransmission: regulation of release and uptake. Basal Ganglia 6:123–148. 10.1016/j.baga.2016.02.001 27141430 PMC4850498

[B86] Tosini G, Menaker M (1998) The clock in the mouse retina: melatonin synthesis and photoreceptor degeneration. Brain Res 789:221–228. 10.1016/s0006-8993(97)01446-7 9573370

[B87] Tsai HC, Zhang F, Adamantidis A, Stuber GD, Bonci A, de Lecea L, Deisseroth K (2009) Phasic firing in dopaminergic neurons is sufficient for behavioral conditioning. Science 324:1080–1084. 10.1126/science.1168878 19389999 PMC5262197

[B88] Turner BD, Kashima DT, Manz KM, Grueter CA, Grueter BA (2018) Synaptic plasticity in the nucleus accumbens: lessons learned from experience. ACS Chem Neurosci 9:2114–2126. 10.1021/acschemneuro.7b00420 29280617 PMC6508969

[B90] Walker DM, Bell MR, Flores C, Gulley JM, Willing J, Paul MJ (2017) Adolescence and reward: making sense of neural and behavioral changes amid the chaos. J Neurosci 37:10855–10866. 10.1523/JNEUROSCI.1834-17.2017 29118215 PMC5678018

[B91] Walker QD, Rooney MB, Wightman RM, Kuhn CM (2000) Dopamine release and uptake are greater in female than male rat striatum as measured by fast cyclic voltammetry. Neuroscience 95:1061–1070. 10.1016/s0306-4522(99)00500-x 10682713

[B92] Walton JC, Weil ZM, Nelson RJ (2011) Influence of photoperiod on hormones, behavior, and immune function. Front Neuroendocrinol 32:303–319. 10.1016/j.yfrne.2010.12.003 21156187 PMC3139743

[B93] Wu Q, Reith EA, Wightman RM, Kawagoe KT, Garris PA (2001) Determination of release and uptake parameters from electrically evoked dopamine dynamics measured by real-time voltammetry. J Neurosci Methods 112:119–133. 10.1016/S0165-0270(01)00459-9 11716947

[B94] Yoest KE, Cummings JA, Becker JB (2014) Estradiol, dopamine and motivation. Cent Nerv Syst Agents Med Chem 14:83–89. 10.2174/1871524914666141226103135 25540977 PMC4793919

[B95] Yorgason JT, España RA, Jones SR (2011) Demon voltammetry and analysis software: analysis of cocaine-induced alterations in dopamine signaling using multiple kinetic measures. J Neurosci Methods 202:158–164. 10.1016/j.jneumeth.2011.03.001 21392532 PMC3149733

[B96] Yorgason JT, Zeppenfeld DM, Williams JT (2017) Cholinergic interneurons underlie spontaneous dopamine release in nucleus accumbens. J Neurosci 37:2086–2096. 10.1523/JNEUROSCI.3064-16.2017 28115487 PMC5338756

[B97] Zachry JE, Nolan SO, Brady LJ, Kelly SJ, Siciliano CA, Calipari ES (2021) Sex differences in dopamine release regulation in the striatum. Neuropsychopharmacology 46:491–499. 10.1038/s41386-020-00915-1 33318634 PMC8027008

[B98] Zhang H, Khan A, Chen Q, Larsson H, Rzhetsky A (2021) Do psychiatric diseases follow annual cyclic seasonality? PLoS Biol 19:e3001347. 10.1371/journal.pbio.3001347 34280189 PMC8345894

